# A Practical Treatise on the Diseases and Injuries of the Urinary Bladder, the Prostate Gland, and the Urethra

**Published:** 1851-10

**Authors:** 


ART. IV.—*A Practical Treatise on the Diseases and Injuries of the Urinary Bladder, the Prostrate Gland, and the Urethra. By* S. D. GROSS, M. D., Professor of Surgery in the University of Louisville, Member of the American Medical Association; author of Elements of Pathological Anatomy, etc. etc. With one hundred and six illustrations. Philadelphia: Blanchard & Lea. 1851. pp. 726. 8vo.

There are few subjects in medical science involved in greater obscurity than the formation of urinary calculi; at the same time it is one which awakens the liveliest interest in the minds of medical men, in a region more especially where the disease is of such common occurrence as it is in the western States. It is a fact now well established that no country of the same population furnishes a larger number of cases of stone than that known as the Valley of the Mississippi. Kentucky, Tennessee, Ohio, and Alabama are said by Dr. Gross to afford a greater number of cases of this disorder than any other portions of the United States. Next to these, are ranked Pennsylvania, Virginia, Maryland, the two Carolinas, Georgia, Florida, Louisiana and Arkansas, while the inhabitants of Missouri, Iowa, Wisconsin, Michigan, Indiana, New York, and New Jersey are comparatively exempt; and in the New England States generally a case of calculus of the bladder is exceedingly rare. Our author is led to believe that the same is true of the Canadas, Texas, Mexico and California.

We suspect that there is some error in this estimate, and that it will turn out that Missouri at least, of the north-western States, has furnished her quota of cases for the lithotomist. Statistics when they come to be collected, we incline to believe, will show that this State ranks quite as high in this respect as Alabama, and higher than the Carolinas, Louisiana, Georgia, Arkansas or Florida. Climate, water, food, and the habits of the people have all been invoked to explain these differences; but up to this time we have to confess that the cause has not been ascertained. What Dr. Gross says of the operation of climate in the formation of urinary calculi is as true of most of the other alleged causes of stone---that their agency is "mysterious." The question is one of great interest, and is at present attracting much attention. Dr. Gross adverts to the opinion that limestone water promotes calculous diseases, and remarks:

"The opinion is plausible, and may be true, but how far, or to what extent, nobody has attempted to decide. If it be true that in Kentucky, Alabama, Tennessee, and Ohio, most calculous cases occur in limestone regions, it is equally true that many are found in the freestone districts of those States."

He might have added, that there is as much limestone in Indiana as in Kentucky, and that it is also an extensive formation in Missouri and the north-western States, in which urinary calculi are said rarely to occur.

About twenty years ago, Mr. Crosse, in an essay on urinary calculus, announced that the great and prevailing source of urinary deposits in the human body is *dyspepsia*, leading to the generation of acid in the stomach and of lithic acid in the urine. It will not be denied that all those articles which have a tendency to create acidity and flatulence, must exert a deleterious influence upon the urinary secretion, changing its properties, and promoting the deposition of earthy matter. But we hold that no one is warranted in saying that, because these articles produce such effects upon the digestive organs they therefore induce calculous diseases; for although dyspepsia prevails extensively in that portion of the United States in which vesical calculus is most frequently observed, it is not less prevalent in Maine, and in the northern States generally, where cases of stone are seldom seen. That the two diseases often, perhaps most generally, exist in the same individual cannot be doubted; but that other causes than disturbance of the digestive functions are necessary to develope the urinary concretion is a fact we think fully established.

Whatever for any length of time, intercepts the free evacuation of the urine favors the formation of stone in the bladder; and hence stricture of the urethra, one of the most common and powerful interruptions, organic disease of the bladder, ureters, and kidneys, and enlargement of the prostate, may be stated as among the most frequent predisposing causes. When an inordinate secretion of mucus is associated with the mechanical obstruction, the liability to stone is greatly increased from the fact, that more or less of the solid constituents of the urine is retained in the bottom of the bladder, where it often becomes the nucleus of a future concretion.

The second section treats of the physical and chemical properties of vesical urinary calculi, and embraces some curious and interesting facts.

Upon our knowledge of the chemical constitution of calculi depends our ability to correct the diathesis and prevent the urinary concretion, and as this knowledge is obtained through the assistance of the chemist, we regret that Dr. Gross has not submitted the many beautiful specimens of calculi in his possession to analysis, and made known the results.

The volume of urinary concretions appears to be in a great degree determined by their chemical composition; and we may consequently, by ascertaining the calculous diathesis of a patient, form a tolerably correct idea of the size of the stone under which he is laboring.

The symptoms of stone are *rational* and *physical*. The rational symptoms are numerous and diversified in character, and are described with great minuteness, and in a manner calculated to enable the merest tyro in surgery to detect, as far as it is possible to do so by rational signs, the presence of a calculus. It is well known however, that these symptoms alone, even though all be clearly present, are not sufficient to impart certainty in such cases. It is only by the physical signs—by the well known, the pathognomonic *click*, caused by the collision of the metallic sound and the stone, and which the contact of no other bodies in the bladder can produce, that the existence of a calculus is converted from a mere probability into a tangible reality. Cases have occurred in the hands of the most experienced and accomplished lithotomists in which, notwithstanding the presence of both the rational and physical signs, calculous disease did not exist; and other cases are reported in which a stone was apparently detected, and yet when the patient was cut, it could not be found. The latter accident has happened on two different occasions in the practice of a surgeon of this city. In the first instance, the patient a little boy,survived, and is still living. The other, an old man, died from the effects of the operation, and on examination, the bladder was found to contain nothing but a fungous tumor, portions of which had repeatedly come away during life. For the purpose of preventing a repetition of such deplorable mistakes, Dr. Gross has indicated their sources, and if his remarks are as carefully studied as they deserve to be, this reproach to surgery will not happen among his readers.

Sounds, the operation, dangers and errors of sounding, latent stone, the symptoms produced by an irritable bladder, and sympathetic irritation and the pathological effects of stone, are the remaining subjects discussed before the author arrives at the treatment of urinary calculi:

"Persons affected with stone do not always find it convenient to submit to the operation of lithotomy, and it

4*

becomes a matter therefore, of great importance to render them as comfortable as their circumstances may admit of."

By appropriate and well directed treatment, by attention to the general health, as regulated by food, drink and exercise, much may be done to allay local suffering, and even in some instances make the patient forget his disease. In reference to this subject—the medical treatment of stone—our author has some practical and judicious remarks. When the urine is decidedly alkaline, acids are generally indicated, and of these Dr. Gross prefers the nitric to any other acid. In certain cases, marked relief follows the use of citric, and tartaric acids, and in others again, phosphoric acid has seemed to exert a better influence. If the urine be acid, alkalis are of course required. The bicarbonates of soda and potassa, exhibited three or four times a day in the proportion of thirty grains to the ounce of strong hop and uva ursi tea produce a most soothing effect in these affections. In fact, whatever be the diathesis or nature of the concretion, Dr. Gross has generally derived most benefit from the use of alkaline remedies, of which he regards soda as superior to any other. The practice of using the acids and alkalis alternately has been followed by better results in his hands than the protracted use of either alone.

The improvement thus produced has in some instances lasted for years, though, generally, it has been of but short duration. Dr. Gross has never attempted either by injections or galvanic electricity to dissolve stone in the bladder. In the section on lithotripsy he pays a just tribute to its inventor, M. Civiale, and expresses surprise that in a country abounding as ours does in calculous diseases this operation should have been so seldom performed, and that so few surgeons should have made it their special study. It is hardly necessary to remark that to crush the calculus is not always practicable, nor is it always desirable. There are cases in which it cannot be accomplished, and others, again, in which it ought not be; and though there are cases in which it is a far superior operation to lithotomy, there are cases in which it is far inferior. The advantages and disadvantages of the operation are fairly exhibited. To the patient, the operation of crushing the stone appears much less formidable than that by the knife, while in reality it is so in but comparatively a small number of cases. Lithotripsy, even when executed by the hand of a master, and upon a choice subject, is obnoxious to serious objections, and may be followed by a number of bad effects; among which we may mention, hemorrhage, rigors and fever, retention of urine, contusion and laceration of the prostate and urethra, cystitis, perforation of the bladder, impaction of the stone in the urethra, peritonitis, and bending and fracture of the lithotriptor.

To this succeeds a section on lithotomy, which we regard as the ablest paper on that subject extant, and of which we propose to give as full an account as our limits will permit.

Of the several methods of lithotomy in vogue in this and other countries, Dr. Gross gives the preference to the lateral section. "I have," he says, "been so much wedded to the lateral method, that I have never felt inclined to employ any other." The wonder to us is, that the whole surgical world does not feel as he does.

The safety of the operation of lithotomy may be said to hinge upon the extent of the incision into the prostate gland. "That the division should always be as limited as it can be conveniently with the safe and easy extraction of the foreign body" is the canon of most modern lithotomists, and one to which our author has never had occasion to regret a strict adherence. Very partially to cut the prostate and to complete the opening by dillitation is stated by Mr. Skey to be "the golden rule." "The wound should in no instance, however bulky the stone may be, extend entirely through the lateral lobe of the prostate." If the stone be unusually large, the opening should be enlarged by incising the opposite half of the gland. "Where the foreign body is of moderate dimensions, incise the gland to a very limited extent, and immediately after enlarge the opening with the finger." When this is not sufficient, "the probe-pointed bistoury is a substitute. It is remarkable how lacerable the organ is in children and adolescents, and to what extent it may be torn, without endangering the parts by infiltration."

Our author holds very different language in relation to the outer wound:

"While the internal should always be small, the external can scarcely be too large, or too free and dependent. The extent of the outer wound should never be less, in the adult, than three inches to three inches and a half; in very young subjects it must, of course, be proportionably limited, but even in them it should rarely be less than two inches and a quarter."

While hemorrhage is of rare occurrence when the pelvis is of the ordinary width, the vessels of the perineum perfectly regular both as respects their distribution and volume, and the proper caution is observed in performing the operation, it occasionally, under an opposite condition of things, becomes one of the most serious accidents attending perineal lithotomy. Dr. Gross does not think with the late Mr. Key and others, that the artery of the bulb—one of the principal sources of hemorrhage—is almost always divided in the lateral operation, "for the reason, that, if this were the case, the mortality from this operation would be much greater than it is."

In order to overcome the difficulty which is frequently experienced in ascertaining whence the blood proceeds, Dr. Gross suggests that a small wire speculum might do good service in the exploration which, under such circumstances, it is always necessary to make.

Our author has no partiality for compression, as a means of arresting hemorrhage, and declares that he never resorts to it but with extreme reluctance.

The circumstances which determine the time consumed in performing the operation of lithotomy, are too well known to require repetition here. One thing, however, in regard to it is settled, to wit: that "no lithotomist should cut against time." The dissecting room rule, of cutting freely so as to effect a rapid operation, and meet with no difficulty to the extraction of the calculus, is not sanctioned by the experienced and practical teacher. The most successful lithotomists have not always been the quickest operators—the quickest operations have not been found the most successful. We once saw Dr. Gross remove a stone in thirty seconds, and we are acquainted with another surgeon who can boast of the same brilliant feat. The surgeon's motto in this, as indeed in all other operations, should and must be, if he would gain many triumphs without the loss of many lives, "*festina lente.*"

Dr. Gross condemns the practice of introducing a tube into the bladder by the wound for the purpose of conducting off the urine, and thereby guarding against infiltration of the cellular tissue, and pronounces it to be not only "useless in reference to the object proposed, but eminently calculated to prove mischievous, and never either necessary or proper except where the incisions have been made unusually extensive." We agree with him in this opinion.

"The treatment after the operation must be strictly antiphlogistic. The patient is kept quietly upon his back, and all excitement, both bodily and mental, is sedulously guarded against. The pain consequent upon the operation is often extremely severe, and is generally referred to the neck of the bladder. It is of a cutting, darting, or burning character, and is either continued, or it comes on in frequent paroxysms. It generally makes its appearance immediately after the operation, and is sometimes so severe as to cause the patient to scream out at the top of his voice. To arrest this suffering as speedily as possible, as well as to allay the general agitation which is so frequent a concomitant of lithotomy, a full anodyne should be administered, generally within a few minutes after the patient has been removed to his bed. If he be an adult, he should take not less than one grain of morphia or its equivalent of solid opium or laudanum. To administer a smaller dose than this would only be temporizing. The pain must be allayed promptly and effectually, otherwise it may be productive of serious mischief, such as exhaustion, convulsions, or inflammation.

"Demulcent drinks should be used freely throughout the treatment, especially during the first few days. They not only allay thirst, but what is of great importance, they dilute the urine, and diminish its acrid qualities, thus rendering it more acceptable both to the bladder and the wound. They may consist of elm-bark water, flaxseed tea, or gum arabic water, and they may be simple, or combined with nitrate of potassa, bicarbonate of soda, or dilute nitric acid, according to the particular indication of each case.

"The diet must be light, unirritant, and of the most simple kind. For the first few days, the patient should take little else than water-panada, thin gruel, or weak chicken broth. After that he may use a little rice, toast and tea, a few crackers, or a small quantity of mush and milk. No meat or vegetables should be permitted under twelve days or a fortnight. It should be remembered that the slightest indiscretion in diet may greatly retard recovery, or even jeopard life. The patient should consider himself as an invalid, and govern his appetite accordingly. Of course, there are exceptions to all rules. Thus, a patient may be in such infirm health as to require a nourishing diet, tonics, and stimulants, from the very commencement of the after-treatment. Much judgment is doubtless required to enable the practitioner to decide when such a plan is proper, and it is obvious that no specific rules can be laid down for his government.

"In all cases I make it a rule to prevent any action of the bowels for the first three days. I have always pursued this practice under a conviction that the less the rectum is disturbed the better it will be for the bladder. For this purpose I invariably give a full anodyne immediately after the operation, even where it may not be necessary on account of the pain and general agitation. Where care is taken to clear out the alimentary canal thoroughly before the operation, the patient cannot suffer any possible inconvenience from the want of an evacuation during the period here specified. At the end of this time I generally order a dose of castor oil, or Epsom salts, assisted, if the purgative is tardy in its action, by an enema of tepid soap-suds. The same, or other means, may be resorted to afterwards to keep the bowels in a soluble condition. If, during the progress of the case, the patient's tongue becomes coated, and his appetite impaired; or if his general health suffers; or he does not improve as well and as rapidly as he ought; or, finally, if the urinary secretion is loaded with mucus and earthy matter; the best remedy he can use is a dose of calomel, which often, in these circumstances, acts like a charm in promoting recovery.

"During the progress of the treatment, it sometimes happens that the edges of the wound become incrusted with earthy matter, forming a thin, whitish sheet, which adheres quite firmly to their surface. The occurrence is not productive of pain; but, as it prevents the formation of healthy granulations, it serves to retard the reunion of the parts, and should, therefore, be promptly attended to. The deposit is of a phosphatic character, and hence the best remedy is the nitric acid lotion, in the proportion of about four drops to the ounce of water, applied by means of a folded cloth. Where the incrustation extends far back, the fluid may be injected once or twice daily into the bladder. In most cases, the local application should be aided by the internal exhibition of the remedy."

Notwithstanding statistics play so large a part in most of the writings of medical men of the present day, Dr. Gross esteems those furnished by the results of the lateral operation so imperfect and unsatisfactory, that he has not taken the pains to collect them. This is to be regretted, for incomplete as these statistics are, they are nevertheless highly interesting, and we should have been gratified, as we are sure his readers generally would have been, if Dr. Gross had given the result of his extensive researches on the subject.

The fact, we believe, is unquestionable, that American surgeons have surpassed all others in this operation.

Much of their success is doubtless owing to the soundness of constitution and pure air enjoyed by the subjects of stone in this country; but something must also be ascribed to their judgment in preparing their patients for the operation and in the subsequent treatment.

Dr. Gross has performed the operation of lithotomy twenty-five times, and without the loss of a patient. A brief history of twenty-four cases is subjoined as a sort of appendix to the article on stone; the twenty-fifth case came under our author's observation while his work was in press. As we have been politely furnished by him with its details we shall include it in our summary.

The subject of every operation was a white male, and aged as follows:—Eleven were under ten years of age, and ranged thus: One of three years and two months; one of three years and a half; one of four years; one of four years and four months; one of five years; two of six years; one of seven years; one of seven and a half years; one of eight years and two months; and one of nine years. Five were between the ages of ten and twenty years, and are set down as follows: One of ten years and six months; two of twelve years; one of fourteen years; and one of seventeen years. Six were between twenty and thirty years of age: One of twenty; three of twenty-two; one of twenty-seven; and one of twenty-eight. The remaining three cases had attained the respective ages of thirty; sixty-five, and seventy-seven years.

The youngest subject operated on was only thirty-eight months old, while the most advanced had nearly reached four score years.

Dr. Gross performed his first operation for stone in February, 1841; and the last in April, 1851. Of the twenty-five cases, Kentucky furnished sixteen; Tennessee, three; Indiana, two; Illinois, one; Arkansas, one; Ireland, one; and Pennsylvania, one. The patient from Pennsylvania was aged twenty-eight years, and had resided eleven years in Kentucky, during the last five of which only had he been affected with symptoms of stone.

The native of Ireland was a lad six years of age. He emigrated to this country when ten months old. After he reached America, he lived one year at Quebec, and afterwards, for twice that period at Pottsville, Pennsylvania. "He was always very healthy till he was two years of age, when he began to suffer from difficulty in voiding his urine, which gradually increased in severity up to the time of the operation." This was performed without accident in June, 1846, and the wound was completely healed by the seventeenth day. "The urine began to pass through the urethra on the fifth day, and on the ninth, it ceased to flow by the wound." Although the patient had a "rapid recovery, he has labored under incontinence of urine ever since the operation. The stone was hardly as as large as a common garden bean, and its weight was only five grains."

Mr. Martineau, of Norwich, England, once removed a calculus of ten grains, which was supposed to be the smallest ever extracted by operation. An ambition of the lithotomist may legitimately be to remove the largest stone, but to subject a patient to the dangers of the knife, and find only a single calculus whose weight may be counted by grains is, in most instances, very properly a source of regret. "If I could have ascertained before hand, in the above case, that the concretion was not larger than it proved to be after removal, I should certainly have performed lithotripsy instead of lithotomy." And so, we presume, would any body else.

The little boy of thirty-eight months old was relieved, in good time, of a concretion hardly as large as a common filbert, and in twenty-four days the wound had completely cicatrized. "For sometime after the operation the patient remained well; but by degrees he became thin and puny, and began to suffer from irritability of the bladder, with frequent pains in the lumbar region. The symptoms gradually increased in violence, and he died in eighteen months from the time he was cut, in a state of excessive emaciation. On dissection, I found the bladder entirely sound and free from calculus deposit; the urethra also was healthy, and so was the right kidney. The left kidney was nearly twice the natural size, and contained a concretion nearly as large as an ordinary hazel nut; the organ adhered firmly to the outer surface of the descending colon, which was perforated by a small opening, the edges of which had a dark, gangrenous appearance."

In the patient aged twenty-seven years, untoward symptoms arose at the end of the third day, in the form of a troublesome diarrhea and an erysipelatous aspect of the wound, which in twenty-four hours after "gave vent to fecal matter, thus showing the existence of an opening between it and the bowel. That this communication was the result wholly of the sloughing process, and not from any improper interference of the knife," is evinced by the fact that Peters "had not less than eleven passages previously without anything issuing at the wound. The patient, whose pulse had become weak and faltering, was promptly put on calomel and opium, with quinine and milk punch, under the use of which he gradually regained his strength; the erysipelas slowly disappeared, and in less than four weeks the wound was entirely healed. When he left town, his general health was greatly improved, and the only inconvenience he experienced was a discharge, at long intervals, of a small quantity of urine by the rectum. I learned, several months after his return home, that the fistula had entirely closed, and that he was enjoying excellent health."

A phosphatic calculus, about the size of a pullet's egg, was extracted from Mr. Orrand, aet 30:

"No accident occurred during the operation; for some days the patient remained weak, and required porter and a nourishing diet, but his flesh and strength gradually increased, and in three weeks the wound was nearly healed, all the urine being voided by the urethra. He went home early in January; for some time after he was much improved, but early in April, 1847, that is, fifteen months from the operation, his calculous symptoms returned, and his sufferings became nearly as great as ever. He now complained of great pain and weakness in the back, and constantly passed calcareous matter. When I last heard from him, his health was very bad, and no doubt existed as to the character of his disease."

The lad, aged four years and four months, had a good recovery, and left Louisville for his home in Indiana, the sixteenth day after the operation. For some weeks he improved very much in health, experienced but little pain in making water, and the wound healed almost entirely. This was early in July. Late in October information came, that the boy had been constantly getting worse for the last two months, and that he suffered quite as badly as before the operation, and serious fears were entertained, that there was a return of the disease. "Whether this was really the case, I am unable to say, as I have since received no further intelligence respecting him."

In three only of the cases operated on by Dr. Gross, was the hemorrhage sufficient to require treatment. The first occurred in a young man twenty years old, who had had symptoms of urinary disease since his childhood, and appeared to proceed from the superficial perineal. "In incising the skin and subcutaneous cellular tissue, I cut an artery, apparently the superficial perineal, which bled so copiously as to require immediate ligation; the blood issued with astonishing impetuosity, and with a peculiar whizzing noise."

In the second case, Dr. Gross was obliged to tie a small deep seated artery, which continued to bleed for some minutes.

The third and last case was that of a man, aged twenty-eight years, in which the same artery was thought to be divided.

"The operation completed, I tied the divided artery, above mentioned, but found to my surprise, that the flow of blood, instead of being arrested, appeared at innumerable points on the surface of the wound. It was of a venous color, and led to the idea that it might proceed chiefly from the neck of the bladder. On cleaning the wound, however, it was found to ooze out, as above stated, at different points. The patient was put to bed in the hope that the hemorrhage would cease, but in this I was disappointed. He was now brought to the edge of the bed, and the wound carefully cleaned, to ascertain if possible, the source of the bleeding. Seeing no vessel from which it could proceed, I determined to introduce a large, soft piece of sponge, sufficiently long to reach the neck of the bladder, and then apply ice to the perinæum and hypogastrium. This had the effect of arresting the bleeding at once. The operation was performed at 10 o'clock in the forenoon; and the next morning at 8, upon removing the plug, the bleeding returned, the blood oozing out as before, and compelling me to reintroduce the sponge. This had again the desired effect; the plug was retained till the following Wednesday, when it was withdrawn, without a recurrence of the hemorrhage. In all other respects, things progressed favorably; there was a little fever for the first two days, and some tenderness in the wound, but nothing serious. It should be observed that the urine flowed freely through the wound by the side of the sponge, for the first twenty-four hours; afterwards most of it flowed along the urethra until the sponge was removed. Nearly three weeks elapsed before it passed through the urethra, and three months before the wound was entirely closed. Both these effects probably depended upon the use of the plug; for his general health was all the time excellent, and could hardly, in my judgment, have had any agency in retarding the healing process."

A remarkably interesting case, in many respects analogous to one already alluded to, is the following:

"Thomas Alexander, fourteen years of age, came to me on the 21st of November, 1849, from Henry county, Tennessee, with symptoms of stone in the bladder. With the exception of two or three mild attacks of intermittent fever, which is common in his neighborhood, his general health was always good until several months ago. He lives in a freestone region, and is not aware that any of his immediate relatives have ever suffered from calculous disease. He has always, from early infancy, had difficulty in voiding his urine, which he has for some time past, been obliged to discharge every fifteen or twenty minutes, often mixed with blood. Within the last six months, he has passed several small concretions, and has suffered much from pain in the sacro-lumbar region. Being in good health upon his arrival in Louisville, I cut Thomas two days after, removing two stones, one of which, shaped somewhat like a squirrel's head, and as large as an ordinary apricot, projected in the neck of the bladder, and was extracted in thirty seconds from the moment the knife touched the integuments. The other was a little smaller, and was also promptly dislodged. No untoward symptoms occurred after the operation. On the 2d of December, the urine passed almost entirely through the natural channel, the wound was nearly closed and the general health was gradually improving. On the 8th, he left for his home in Tennessee, in excellent condition, the opening being almost cicatrized, and permitting only a few drops of urine to escape through at a time. He was directed to live light, to pay attention to his bowels, and use an infusion, three or four times daily, of uva ursi and hops with bicarbonate of soda.

Soon after reaching home, Thomas was taken worse; there was a return of pain in the bladdder, and he had great, and incessant distress in the region of the kidneys; with violent tenesmus, and a frequent desire to micturate; in short, he had again all the symptoms of urinary calculus. The general health gradually declined, and the wound never entirely healed, owing to the constant passage of sabulous matter, and the adhesion of more or less of this to the sides of the opening. Every thing was done to mitigate his suffering, but to little purpose. Nothing but the most liberal use of anodynes afforded the slightest relief. In this condition, his father determined to put him on board a steamer, and revisit Louisville; he arrived in town on the 3d of October, 1850. Soon after leaving home, Thomas was seized with vomiting and purging, followed in forty-eight hours, by symptoms of dysentery. He was unable to retain anything on his stomach; his thirst was excessive; and he had frequent discharges of blood and mucus. He had improved somewhat in flesh during the last two months; but since this attack he had become greatly emaciated. The case resisted all treatment, and he expired early in the morning of the 5th. A careful examination revealed the following facts.

"The fistula in the perinæum was of the diameter of a common probe, and was incrusted, nearly in its entire extent, by sabulous matter, a small quantity of which was visible at its external orifice, situated at the upper part of the cicatrice. The skin for some distance around the opening was red and excoriated, from the acrid nature of the discharges.

"The bladder was much contracted, and hardly capable of holding a pullet's egg; it contained no urine, but some purulent matter, and fifteen calculi, from the size of a mustard seed to that of a pea, irregular in their shape, of a white color, and rough on the surface. The mucous coat was considerably thickened, mammillated, and throughout of a slate color. Sabulous matter existed in abundance at the bottom of the vesical orifice of the fistula, and readily accounted for the want of union of the wound. The coats of the bladder were pale, firm, and nearly half an inch thick in the posterior half of the organ; but in front of this they were free from hypertrophy and and of the natural consistence. The urethra was healthy. The prostate gland was remarkably small, but of normal color and consistence.

"The ureters were of a pale drab color externally, unusually firm and large, and remarkably thickened in their coats; they contained each a small quantity of muco-purulent matter, intermixed with gritty substance. Their lining membrane was longitudinally corrugated, of a pale straw color, and considerably thickened. The renal extremities of both tubes were somewhat contracted.

"Both kidneys adhered with great firmness to the surrounding parts, and were imbedded in a small quantity of at. The right was somewhat hypertrophied, hard, and of a dusky hue externally; internally it was more pale; the calyces were enlarged, and filled with purulent matter and grains of sand, with several small concretions similar to those found in the bladder. The left kidney was a little larger than the right; when pressed upon it grated, and felt like a bag of mortar; it was remarkably firm, and of a dusky color, and contained a large quantity of sabulous and stony matter. The mucous membrane of the pelvis and calyces was chronically inflamed. The right renal capsule was natural; the left had disappeared. The abdominal viscera were sound. The rest of the body was not examined."

It is a frequent subject of remark, that urinary calculus is a disease of such very rare occurrence among the African race. Of the two hundred individuals lithotomised by Dr. Dudley, only six are reported to have been negroes, and as we have elsewhere stated, all the subjects of Dr. Gross's operations were whites. We have received information of two cases of this affection in negroes; one living in Tennessee, and the other in this State.

The three examples which constitute the only approach among the whole number to anything like hereditary predisposition to stone are the following:—A man whose father and paternal uncle both had stone—an individual, whose maternal grandmother had stone—another, two of whose maternal uncles had voided small calculi on different occasions.

It was also in only three of the twenty-five cases, that Dr. Gross found more than a single concretion. From the patient aged 77, he extracted "fifty-four calculi, varyrying in size from that of a pea to that of a partridge egg. All, excepting one, seemed to be encysted at the superior fundus of the bladder, which was immensely elongated, extending nearly to a level with the umbilicus. The forceps and scoop were introduced at least thirty times, and not a little difficulty was experienced in rupturing the cyst and detaching the calculi." The second case—that of Thos. Alexander, has been already alluded to. From the third patient, operated on in April last, Dr. Gross removed t'ree calculi, two situated in the urethra, the third occupying the bladder.

In some of the earlier cases recorded by our author, he has omitted to state the geological formation of the district of country in which the patients resided. After supplying this deficiency, we find that seventeen cases were from a limestone region; six lived in a sandstone region; one—the case from Pennsylvania, had used for several years before the earliest symptoms of stone were made manifest, the water of the Ohio and Mississippi rivers. The Irish boy had lived in Quebec, Pennsylvania, and Louisville, Kentucky. We have not been able to gather from his history as reported, whether or not he was living at the time of his first attack in a lime or a sandstone region. He had no symptoms of calculous disease however, until he had been about fourteen months in the United States.

The remaining sections of this chapter are devoted to a description of the several other modes of extracting urinary calculi, and are replete with very interesting details.

The first part of the work is concluded by a chapter on foreign bodies in the bladder. Part second embraces the *diseases and injuries of the prostate gland.* The various accidents and affections to which this gland is liable are treated of with the same fidelity which has characterised those portions of the work of which we have already spoken. Hypertrophy, abscess, and calculi of the prostate are illustrated by a number of striking figures. Part third is devoted to the *diseases and injuries of the urethra.* While speaking of stricture of the urethra, our author inculcates some principles which we commend to the attention of our readers:

"B.fore resorting to any of these expedients,"—dilatation, compression, cauterization, &c. &c., "it is of paramount importance, I conceive, to attend to the general health, and to subdue local inflammation. Unless this be done, the practitioner will run great risk of doing harm instead of good. To effect this object, the patient should be kept in the recumbent posture for six or eight days previously to the intended operation; the bowels should be freely moved every forty-eight hours with some mild purgative; the secretions should be duly regulated; the diet should be light and unirritant; and recourse should be had occasionally to the warm bath. If there be any inflammation, irritation, or spasm of the urethra and the bladder, leeches must be applied to the perinæum, followed by fomentations and anodyne enemata. Demulcent drinks should also be used; and there are few cases which will not be benefited by the exhibition of bicarbonate of soda and balsam of copaiba. Too much stress cannot be placed upon this preliminary treatment; indeed, I should consider it highly culpable to neglect it under any circumstances. When the way has been thus paved, the particular kind of treatment is to be determined by a careful consideration of the nature of the obstruction. There are few points in surgery which require more judgment and experience than this.

"Some practitioners are in the habit of relying mainly, in their attempts to cure organic stricture, upon constitutional means, especially rigid abstinence, carried almost to starvation, and the daily use of nauseating doses of tartarized antimony, or the frequent exhibition of emetics; conjoined with rest in the recumbent posture, and the avoidance of all sources of bodily excitement. That such a mode of treatment is well calculated to allay vascular action, and promote the absorption of the effused lymph which gives rise to the obstruction, may be readily imagined; but any advantages thus accruing are generally more than counterbalanced by the hardships which attend it. In the callous form of the disease, such a proceeding must be perfectly futile; for there are few cases which can receive any permanent benefit from it, and in which it will not be more likely to wear out the patient than his stricture. Of a considerable number of persons whom I have known to be treated upon this principle, I do not recollect a single one that experienced any decisive or permanent relief, or that was willing again to submit to its exorbitant, unscientific, and injurious exactions."

Of dilatation he says:

"My conviction, founded upon ample experience, is, that the very best instrument for dilating a stricture is the common silver catheter, with a slightly conical point. I have employed this instrument now in the treatment of this affection for upwards of twenty years, and nothing could induce me to abandon it. It possesses all the requisites that such an instrument ought to have, namely, lightness, firmness, and durability, and is incomparably superior, in every respect, to all the metallic, wax, gum-elastic, ivory, and other bougies that have ever been invented. In making this statement, I do not wish to be understood as saying that bougies are entirely worthless, or that they ought never to be used. If I were to do this, I should certainly do injustice to my judgment, and assert what is contrary to the experience of some of the ablest and most enlightened surgeons of the age, both in this country and in Europe. My desire is merely to recommend, in strong and decided terms, an instrument which is capable, as a general rule, of fulfilling every indication presented in this disease, even in its worst forms, and which, I am satisfied, is much less appreciated than it deserves to be."

"Cauterization, properly used, is a valuable curative agent; but, if indiscriminately employed, it is capable of doing serious, if not irreparable mischief. The circumstances to which it appears to be more particularly adapted, and to which, in my judgment, it ought to be restricted, are those in which the stricture, without being very tight or extensive, is of a firm, gristly, and resilient character, and in which there is an undue amount of morbid sensibility of the mucous membrane of the urethra. I never resort to it where the obstruction is either very slight or very great; for in the former case I have rarely failed to effect a cure by dilation, and in the latter I have almost always been obliged to have recourse to its division. It may be further observed that cauterization should seldom be ιelied upon alone, but that its action should always be aided by the bougie or catheter; a mode of proceeding which greatly expedites the cure, and affords an additional guarantee against relapse, which is so apt to occur when this precaution is omitted."

"The method by incision, as recommended and practised by Mr. Stafford and other surgeons has met with much opposition, and has even been denounced as barbarous and disgraceful. In such an opinion I cannot concur.

I have performed the operation too frequently not to be convinced of its efficacy, if not of its superiority over all other plans.

"When a stricture is very old, firm, and unyielding, or almost cartilaginous in its consistence, no mode of dilatation, however judiciously and perseveringly employed can succeed, either alone or in combination with cauterization, and in such a case I never hesitate to resort at once to incision, satisfied that nothing else will answer. I have repeatedly had under my charge patients who had been subjected to the treatment by dilation for months and months, without the slightest benefit, and who were almost instantly relieved by the operation under consideration. This fact has been witnessed again and again by my private pupils and by the public classes of the University of Louisville. And why should there be any hesitation or doubt concerning this operation? Where are its dangers, or the difficulties of its execution? I confess I cannot see any; and in making this remark let no one regard me as a visionary enthusiast. What I say is not speculation, but the result of personal observation; not prejudice, but actual experience at the bedside. It is only when the stricture is situated far back, in the membranous portion of the urethra, that the method is obnoxious to objection. Under such circumstances, especially when the obstruction is nearly impermeable, or where it is accompanied by a tortuous condition of the urethra, there may, I admit, not only be danger in attempting division, but the operation requires an amount of skill and anatomical knowledge which few men possess. But even here the well-directed efforts of the patient and persevering surgeon will generally be crowned with success. To the unskilful alone is the operation a stumbling-block; to the ignorant, foolishness."

"Scarification, as a remedy for the cure of stricture, acts in the same manner as incision, of which, as was before intimated, it is merely a modification. The process is best adapted to the milder varieties of firm, gristly contractions, seated in the spongy portion of the canal. I should certainly not resort to it either in the very simple or very aggravated forms of the malady; in the first it would be unnecessary, in the second, inadequate."

But we must bring our article to a conclusion. It is long, but we believe our readers will not deem it too long, considering the interest of the subjects treated of, and the ability with which they have been discussed in the treatise before us. Dr. Gross has brought all his learning, experience, tact, and judgment to the task, and has produced a work worthy of his high reputation. We feel perfectly safe in recommending it to our readers as a monograph unequalled in interest and practical value by any other on the subject in our language; and we cannot help saying, that we esteem it a matter of just pride, that another work so creditable to our country has been contributed to our medical literature by a western physician. This treatise will take its place by the side of those of Professor Drake and Professor Miller, which have passed into the very first rank of American productions. It is a little more than two years since we had the pleasure of calling the attention of our readers to the original and judicious work of Prof. Miller on Parturition, which, as they are aware, has since been republished in London. The elaborate and profound work of Prof. Drake was announced about a year ago, and by the medical press at home and abroad, has been pronounced one of the ablest ever written by an American physician. We doubt whether more valuable accessions to our professional literature have been received in the same time from any quarter.

